# A Chapter on Cholera for Lay Readers

**Published:** 1893-11

**Authors:** 


A CHAPTER ON CHOLERA, FOR LAY READERS—HISTORY, SYMPTONS AND TREATMENT OF THE DISEASE—By Walter Vought, P. H. B., M. D., Medical Director and Physician in charge of the Five Island Quarantine Station, Port of New York; Fellow of the New York Academy of Medicine, Etc. Illustrated. Published by F. A. Davis & Co., Philadelphia and London. 1893.

Dr. Vought has given us in a few pages a very concise and interesting discourse on cholera. Not only the layman, but the physician will profit by reading it, for the newest and most approved ideas are suggested and advised.

This work is especially valuable, as it is the expression of a close observer and ready student, who, as a digest of his personal experience, and that experience large, gives us the knowledge so acquired.

We take pleasure in recommending this work

